# Safety considerations and risk mitigation strategies for ketamine use: a comprehensive review

**DOI:** 10.1097/MS9.0000000000003232

**Published:** 2025-04-02

**Authors:** Reem Sayad, Ahmed Saad Elsaeidy, Amna M. Anis, Mohamed Atef, Eslam A. Hawash, Hager Adel Saad, Khadijah Ali Abdalrahman Hamad, Neveen A. Kohaf

**Affiliations:** a Faculty of Medicine, Assiut University, Assiut, Egypt; b Faculty of Medicine, Benha University, Benha, Egypt; c College of Medicine, Alfaisal University, Riyadh, Saudi Arabia; d Faculty of Pharmacy and Biotechnology, German University in Cairo (GUC), New Cairo, Egypt; e Faculty of Dentistry, Alneelain University, Khartoum, Sudan; f Department of Clinical Pharmacy, Faculty of Pharmacy (Girls), Al-Azhar University, Cairo, Egypt; g Department of Clinical Pharmacy, Faculty of Clinical Pharmacy, Al Baha University, Al Baha, Saudi Arabia

**Keywords:** anesthesia, depression, ketamine, pain, safety

## Abstract

Ketamine, a dissociative anesthetic, has gained widespread use in various medical contexts, including anesthesia, pain management, and treatment-resistant depression. Despite its therapeutic potential, concerns regarding its safety profile have prompted ongoing research and regulatory guidance. This comprehensive literature review explores the current safety considerations of ketamine, summarizing its adverse effects, particularly on cardiovascular, neuropsychiatric, and dependency effects. Evidence-based guidelines for its administration, dosing, and monitoring are discussed, emphasizing the importance of risk-benefit assessments in clinical practice. The review also delves into current guidelines and proposes future directions for ketamine research and clinical implementation, including enhanced safety protocols, long-term patient outcomes, and the development of individualized safe dosing protocols.

## Introduction

Ketamine, an NMDA receptor antagonist introduced in 1964, was originally developed as an anesthetic (1). It is commonly used in anesthesia due to its dissociative properties, which result from NMDA receptor blockade at high doses. This dissociation allows patients to remain conscious while being detached from sensory perceptions^[2]^. Ketamine is also effective in pain management, with proven analgesic benefits for both acute and chronic pain. Low intravenous doses of ketamine have shown comparable analgesic efficacy to IV morphine for acute pain but are typically combined with other pain medications. Postoperatively, ketamine significantly reduces pain intensity and opioid requirements^[3-5]^.

**HIGHLIGHTS**

- Ketamine’s dissociative effects make it a substance with a potential for misuse and dependence.
- Careful cardiovascular monitoring during use, particularly in patients with preexisting hypertension or cardiac conditions, to prevent adverse events is necessary.
- Adhering to recommended dosing guidelines of ketamine and monitoring respiratory status during administration is essential for preventing complications.
- Risk management strategies include patient education, screening for a history of substance use disorders, and regulating access to ensure therapeutic use is controlled.
- Risk mitigation includes pre-treatment counseling, using lower doses, and ensuring a safe, monitored environment during administration.

Ketamine is now widely used for psychiatric conditions like major depressive disorder (MDD) and suicidal ideation, showing rapid improvement in patients with treatment-resistant depression^[6,7]^. Clinical trials confirm ketamine’s effectiveness in reducing depressive symptoms, though its impact is often transient, lasting one to two weeks after infusion, with limited long-term effects reported^[8,9]^.

However, prolonged use can lead to cognitive impairments, including memory and concentration issues, as well as tolerance and physical or psychological dependence. These risks have led to ketamine being classified as a Schedule III controlled substance in the U.S.^[10]^.

Before administering ketamine, psychiatrists should assess the patient’s psychiatric history and screen for heart conditions, as ketamine can worsen issues like hypertension and arrhythmias^[11]^. Continuous monitoring of oxygen saturation and vital signs is crucial to ensure patient safety and hemodynamic stability^[12]^.

Given the safety concerns and risks, including its potential for abuse, healthcare providers must follow strict guidelines to ensure ketamine’s safe use in clinical settings^[13]^. Healthcare providers need to apply these guidelines to ensure the safe and effective use of ketamine in clinical practice^[12]^. This review will cover safety considerations and risk mitigation strategies to minimize adverse effects and promote recovery. These strategies include selection criteria, monitoring requirements, dose adjustments, and measures to prevent misuse.

## Method section

A comprehensive literature search was conducted to identify relevant studies, clinical guidelines, and reviews related to the safety considerations and risk mitigation strategies for ketamine use. The search encompassed multiple electronic databases, including PubMed, Scopus, Web of Science, and Google Scholar. The search strategy employed a combination of Medical Subject Headings (MeSH) terms and free-text keywords, such as “ketamine safety,” “adverse effects of ketamine,” “ketamine monitoring strategies,” “ketamine abuse potential,” and “clinical guidelines for ketamine use.”

Studies were included if they met the following criteria:^[1]^ Published in peer-reviewed journals between 2000 and 2024.^[2]^ Focused on ketamine use in anesthesia, pain management, or psychiatric treatment.^[3]^ Addressed safety concerns, adverse effects, or risk mitigation strategies.^[4]^ Clinical trials, observational studies, systematic reviews, or clinical guidelines were included.

## Adverse effects and safety concerns

### Adverse effects of ketamine use

The effects of ketamine can be felt minutes after usage. Depending on how much of the drug is taken, a variety of adverse effects may be experienced, varying in intensity. Some describe effects that last for several days, even if the acute effects might only last for a few hours^[14-16]^.

Ketamine has adverse pharmacological reactions that affect several body systems. It causes allergic reactions such as anaphylaxis and angioedema^[17,18]^. Injection site reactions, erythema, localized pain, and morbilliform rash are dermatological responses of ketamine administration^[19]^.

It also affects the cardiovascular (CV) system; it may cause temporary elevations in blood pressure, bradycardia, left ventricular dysfunction in heart failure patients, respiratory and cardiac arrest, and arrhythmias^[20,21]^. It also affects the gastrointestinal (GI) system, causing anorexia, nausea, and vomiting. Ophthalmic and respiratory systems are other systems that are also affected by ketamine administration. It causes diplopia, nystagmus, and elevated intraocular pressure^[22]^. Apnea, heightened laryngeal and tracheal secretions, laryngospasm, airway blockage in babies (perhaps non-drug-related), and respiratory depression associated with ketamine use^[23]^.

Ketamine also produces neuromuscular effects. It causes muscle rigidity, spasms, or tonic-clonic movements similar to seizures, as well as increased skeletal muscle tone^[24]^. Additionally, it causes confusion, emergence, and delirium. Ketamine also has a psychiatric effect such as amnesia, anxiety, sadness, disorientation, dysphoria, dissociative states, unusual thoughts, intense fear, exhilaration, illogical behavior, and sleeplessness^[25]^.

### Potential for abuse and dependence

Although ketamine shows promise in treating various diseases, its psychoactive side effects and abuse potential limit its widespread use^[26]^. Recreational doses are about 15–20% lower than anesthetic doses, and its anesthetic and reinforcing effects contribute to its misuse globally. A 2006 U.S. survey reported that 2.3 million adolescents and adults had used ketamine at least once^[24]^. while in 2015, this figure increased to 3 million (1.1%) for individuals aged 12 and older^[27]^. However, recent 2020 statistics show a decline in ketamine misuse, with 1.1 million Americans using it as a hallucinogen^[28]^.

The incidence of ketamine-related fatalities in the UK increased tenfold from 1999 to 2008^[1]^. An Australian survey found that 40% of party drug users reported using ketamine^[29]^. Over the last decade, ketamine addiction has surged in various regions of Asia, with Malaysia experiencing a fourfold rise in users between 2006 and 2012^[30]^. Ketamine misuse first emerged in China during the 1990s, particularly affecting Hong Kong, which recorded over 2000 cases in 2013 and 2014^[31,32]^. In mainland China, the proportion of ketamine users among all reported drug users rose from 21.5% in 2001 to 40% in 2009^[33]^.

The rising severity of ketamine misuse has led to its reclassification from a Schedule II to a Schedule I psychoactive substance in China, despite its relative safety in medical contexts^[1]^. Ketamine’s popularity as a club drug raises concerns about driving under its influence; in Shanghai, it ranked as the third most common illicit substance found in drivers^[34]^. A Scottish survey indicated that 36% of partygoers admitted to driving after using ketamine, and 9% of drivers involved in fatal accidents in Hong Kong tested positive for the drug^[35]^. Ketamine may impair executive cognitive functions, attention, and memory, increasing the risk of road accidents^[27,36]^. Additionally, ketamine is linked to heightened sexual experiences, raising concerns about drug-facilitated sexual assault^[37]^. The growing prevalence of risky sexual encounters, particularly among gay males associated with ketamine use, has prompted alarm in multiple countries^[38-40]^.

Recently, North American print media have shifted to portraying ketamine as a therapeutic antidepressant rather than a substance of abuse^[41]^. However, increasing cases of urological toxicity linked to ketamine misuse have led to usage restrictions in certain regions^[34,42]^. While intranasal (IN) esketamine is approved for treatment-resistant depression (TRD), regulations vary by country. In Canada and the U.S., esketamine is available through regulated distribution programs, such as the JANSSEN JOURNEY™ Program and the Risk Evaluation and Mitigation Strategy, requiring healthcare professionals to supervise and monitor patients for at least two hours post-administration^[43,44]^.

## Monitoring and managing ketamine-related adverse events

### Strategies for monitoring adverse events

Both before and after ketamine administration, monitoring CV, neuropsychiatric, and GI markers is critical for managing treatment-emergent adverse events (TEAEs). Adverse event monitoring strategies include pre-treatment physical and mental examinations, continuous CV monitoring, and patient supervision for neuropsychiatric symptoms such as dissociation and psychotomimetic effects^[45]^. Furthermore, GI problems can be addressed by fasting before administration, using antiemetics, and educating patients about typical side effects, with emergency measures in place for extreme responses^[46,47]^.

Ketamine administration necessitates strict monitoring of respiratory function, as it can produce respiratory depression or apnea, particularly at larger doses used for sedation^[48,49]^. Continuous pulse oximetry is indicated for detecting hypoxemia early^[50]^. In cases of severe respiratory compromise, extensive airway treatment, including endotracheal intubation, may be required^[51]^. Parks *et al* reported a significant intubation rate of 16.3% among 86 patients treated for acute agitation or agitated delirium in prehospital settings, particularly following the administration of high-dose intramuscular (IM) ketamine^[52]^. If respiratory distress is identified, non-invasive ventilation procedures such as bi-level positive airway pressure (BiPAP) or continuous positive airway pressure (CPAP) might be used as a barrier before resorting to more intrusive measures like intubation^[53,54]^. This method can stabilize the patient while minimizing the risks associated with intubation^[55]^.

Johnston *et al* explored various airway management techniques, particularly in the context of emergency medical services (EMS) and sedation with ketamine^[56]^. Key techniques included BiPAP: a non-invasive ventilation method that provides two levels of pressure to assist patients with respiratory distress. CPAP is another non-invasive method where constant pressure is applied to keep the airways open. It is primarily used in cases like obstructive sleep apnea and acute respiratory distress. Moreover, advanced airway monitoring techniques such as endotracheal intubation (ETI) and supraglottic airway devices (SAD) are utilized for securing the airway during sedation with ketamine^[56,57]^.

Integration of these techniques is critical during ketamine administration, particularly in emergency settings. As per the sedation protocol, ketamine is often administered at a dosage of 0.5 mg/kg intravenously for sedation, which necessitates effective airway management due to its respiratory depressant effects^[56]^. A second-generation SAD is preferred for airway management, allowing for quicker and more reliable airway control compared to traditional methods like ETI. All airway interventions require monitoring of end-tidal carbon dioxide (ETCO2) to confirm effective ventilation and oxygenation^[56]^. Introducing rocuronium (1.5 mg/kg IV) after a failed SAD attempt allows for paralysis, increasing the chances of successful intubation if necessary^[58]^. Post-implementation reviews of airway management techniques indicate a higher success rate when protocols are followed, demonstrating the effectiveness of advanced techniques in real-world scenarios. The adoption of protocols that emphasize SAD usage and robust monitoring can significantly improve patient outcomes^[59,60]^. The main monitoring indicators, possible side effects, and management techniques for adverse occurrences both during and after ketamine administration are listed in the table below (Table 1).

**Table 1:** Critical variables requiring monitoring during and after ketamine treatment

Parameter	Potential adverse events	Monitoring method	Intervention/strategy
Respiratory function^[45-47,53,61,92]^.	Hypoxia, respiratory depression.	Continuous pulse oximetry.	Advanced airway interventions (endotracheal intubation, BiPAP, CPAP).
		Observation of the need for advanced airway interventions.	Immediate availability of equipment and a qualified ED physician.
Cardiovascular parameters^[45-47,53,61,92]^.	Hypertension, hypotension.	Continuous blood pressure monitoring.	IV antihypertensive medications, and fluids for hypotension.
	Dysrhythmias.	Continuous heart rate monitoring.	Continuous cardiac monitoring and management of new dysrhythmias.
Neuropsychiatric status^[45-47,53,61,92]^.	Mood changes, dysphoria, confusion.	Regular assessment of mental status.	Continuous observation, supine positioning with head elevation, and protocol reminders.
	Hallucinations.	Observation for hallucinations.	Avoid administration in patients with histories of psychosis; regular staff education.

CV function should be constantly monitored to determine the necessity for advanced airway treatments such as endotracheal intubation or non-invasive breathing systems like BiPAP or CPAP, continuous pulse oximetry, blood pressure, and heart rate. CV indicators should be monitored with special attention paid to any major changes that necessitate treatments such as IV antihypertensive medicines or the care of new dysrhythmias^[46,47]^. Continuous cardiac monitoring, supine patient placement with head elevation, prompt access to advanced airway equipment, and having a skilled emergency department physician are all strategies for mitigating these hazards^[45]^. Additionally, ketamine can produce mood changes, dysphoria, disorientation, and hallucinations; hence, it is important to check neuropsychiatric conditions^[45,61,62]^.

Concerning its distinct antidepressant profile, healthcare practitioners must employ monitoring techniques and protocols for ketamine-related side effects. The Ketamine Side Effect Tool (KSET) is a complete system built specifically for this purpose, with modules for screening, baseline assessment, acute therapy, and follow-up to measure both immediate and long-term adverse effects^[63]^. To address issues including momentary dissociation, hypertension, and nausea, practitioners should educate patients, maintain a calm treatment atmosphere, and be ready to change doses or deliver more drugs as needed. Specific tools such as the clinician-administered dissociative states scale (CADSS) are used to assess dissociation levels^[64]^.

In their study, Mo *et al* used two protocols: sub-dissociative-dose ketamine (SDDK) for pain relief, and higher doses for dissociative sedation in extreme agitation/excited delirium. SDDK was supplied intravenously at 0.2-0.3 mg/kg, with a maximum dose of 25 mg, and can be repeated after 30 minutes. For severe agitation, a dose of 4 mg/kg IM up to 500 mg was administered. Vital signs, continuous pulse oximetry, and telemetry were monitored for 30 minutes after administration, with advanced breathing equipment available right away and continuous and direct observation. The protocols sought to reduce major respiratory, CV, and neuropsychiatric adverse effects, such as intubation, hypertension, and mood swings. The difference between the two protocols is discussed in Table 2. Using SDDK for analgesia, dissociative sedation, and acute excitation resulted in fewer major respiratory adverse events compared to earlier work^[53]^.

**Table 2:** Summary of protocols: SDDK for analgesia versus dissociative sedation ketamine for severe agitation/excited delirium in the ED^[53]^

Protocol^[53]^.	Subdissociative-dose ketamine (SDDK) for analgesia	Dissociative sedation ketamine for severe agitation/excited delirium
Indication	Analgesia for severe pain, including the following:	Severe agitation/excited delirium (pharmacologic monotherapy for adult patients), meeting the criteria:
	Traumatic injury-induced acute pain Recorded parenteral opioid therapy intolerance’s acute pain Chronic pain patients, not candidates for opioid or NSAID therapy	
		Immediate threat to patient and healthcare provider safety (RASS score of +4) Alternative non-pharmacologic de-escalation strategies’ failure and/or futility IV access absence Not a candidate for IM antipsychotics and/or benzodiazepines
Dose	0.2-0.3 mg/kg IV (maximum 25 mg)	4 mg/kg IM (maximum 500 mg)
Administration	Intravenous (IV)	Intramuscular (IM)
Repeat dosing	May be repeated after 30 minutes.	Typically, a single dose; reassess as needed.
Monitoring requirements	Vital signs (including pain assessment) at baseline, 15 minutes, and 30 minutes after each dose	Continuous direct observation for at least 15 minutes
		Continuous pulse oximetry, cardiac monitor, and end-tidal CO2 monitoring (if available)
	Continuous pulse oximetry for at least 30 minutes after dose administration	
	Telemetry for 30 minutes post-administration	Removal of physical restraints Supine patient positioning, with bed head elevation at 30°
	Immediate availability of ED attending physician for at least 30 minutes	
Adverse event considerations	Unstable vital signs	None
	Systolic blood pressure >180 mmHg Heart rate >150 beats per minute. Respiratory rate: <10 or >30	
	Respiratory depression, CV changes, and mood alterations	

### Patient selection criteria and assessment of comorbid conditions

#### Selection criteria for patient selection based on medical history and current medications

Before administering ketamine, it is crucial to review CV safety guidelines and existing medications to identify any contraindications or increased risks of adverse effects^[55,65]^. A review by Meshkat *et al* identified three pharmacogenomic predictors affecting ketamine’s clinical efficacy and side effects: the Val66Met (rs6265) brain-derived neurotrophic factor (BDNF; Met allele), linked to reduced antidepressant effects; CYP2B6*6 (CYP2B6 metabolizer), associated with stronger dissociative effects; and the NET allelic (rs28386840) variant, linked to serious CV complications^[58]^.

To enhance therapeutic outcomes, patient selection criteria should incorporate these genetic predictors alongside a comprehensive medical history assessment, mental evaluation, and review of current medications, focusing on hepatic activity, abuse potential, and existing CV conditions. Monitoring should extend to vital signs, cognitive function, and genitourinary health, with assessments for misuse risk^[11,45]^. Prior to each ketamine infusion, it is essential to reassess new health issues and physical symptoms. Laboratory tests such as a complete blood count (CBC), basic metabolic profile, liver function tests, thyroid stimulating hormone (TSH) levels, and an electrocardiogram are vital for medical clearance, with additional tests for vitamin D, folate, and inflammatory cytokines^[66]^.

Eligibility criteria typically include treatment-refractory non-psychotic unipolar or bipolar depression and other psychiatric conditions where ketamine has shown effectiveness, such as suicidality^[67,68]^. Exclusion criteria involve significant vascular aneurysms, psychotic symptoms, limited cognitive capacity to comprehend therapy risks and benefits, and current substance abuse. Due to ketamine’s potential cardiac effects, a physical examination is necessary to ensure stable vital signs. The American Society of Anesthesiologists Classification (ASA Class) system can help identify individuals who require formal anesthesia consultation, particularly those classified as ASA category 3 or above^[66,69]^.

#### Assessment of comorbid conditions that may affect ketamine safety

Patients suffering from chronic pain or those taking opioids prior to surgery may react differently to ketamine, needing careful assessment and monitoring^[70]^. In addition, comorbid psychiatric problems should be examined since they might influence the frequency of ketamine-related adverse events such as hallucinations or visual abnormalities^[71,72]^.

An editorial study on IV ketamine’s safety in TRD subjects determined that dissociative and psychotic symptoms typically peaked 30 minutes after infusion and resolved within 60 minutes, emphasizing thorough assessment and close monitoring of patients with comorbid diseases, particularly epilepsy^[65]^. In addition to concurrent drugs, mental and medical comorbidities must be considered to properly manage any adverse effects.

## Training and education for healthcare providers

### Importance of provider training

Ketamine has gained widespread use in treating mood disorders, acute pain, and chronic pain; however, caution is essential to achieve desired outcomes and prevent complications, including addiction from misuse by clinicians or patients^[36]^. In 2015, the World Drug Information Centre reported that ketamine was used recreationally in 58 countries^[73]^. To promote effective ketamine use, various psychiatric and anesthesiology institutions, such as the Integrative Psychiatry Institute (IPI) and the Ketamine Research Institute, offer courses and training for clinicians on best practices^[74-77]^.

The Integrative Psychiatry Institute (IPI) offers online training in collaboration with the American Society of Ketamine Physicians, Psychotherapists, & Practitioners (ASKP3), featuring over 15 experts who teach six modules on evidence-based ketamine treatment for mood disorders and pain, administration routes, dosing, psychedelic medicine, post-administration care, and drug interactions^[74]^. Additionally, the Ketamine Research Institute provides an intensive training course that emphasizes evidence-based medicine and hands-on clinical experience, aligning with recommendations from the American Psychiatric Association and the American Society of Anesthesiologists, as well as new safety regulations for administration^[77]^.

The ketamine-assisted psychotherapy initiative provides an advanced program for clinicians in 12 online classes with video demonstrations, peer learning experience, case-based learning series, screening, and practice materials^[75]^. Ketamine Academy offers two types of training programs. Both teach you all the necessary information to provide adequate and safe subanesthetic ketamine therapy^[68]^. The difference between these three programs is summarized in Table 3.

**Table 3:** Summary of differences among the three provider training programs

Name of program^[74-77]^	Price	Content
Integrative psychiatry institute	Full tuition: $8000	Basic, science, clinical practice, using with other therapies, business, maintenance, and aftercare.
	The full discount of $7500 was paid.	
Ketamine research institute	$8950	The current state of using ketamine, efficacy versus effectiveness, and the precision medicine approach.
Ketamine-assisted psychotherapy	$5500	Basic orientation, ethical consent, ketamine psychology and neuroscience, preparation in practice, preparation for the ketamine session, protocols, and therapy, ketamine in dosing session, integration of three sections, business of ketamine therapy, putting ketamine therapy into practice.
	Students can pay three equal monthly payments of $1925 for a total of $5755.	

In addition to training programs for optimal ketamine use, a study highlighted effective strategies for treating resistant depression, generalized anxiety disorder (GAD), obsessive-compulsive disorder (OCD), suicidality, post-traumatic stress disorder (PTSD), and substance use disorder among healthcare providers (HCPs), who are more susceptible to these conditions due to their work^[68]^. One effective approach involves ketamine-assisted therapy (KAT) administered at doses of 1 to 1.5 mg/kg via intramuscular injection, combined with a Community of Practice (COP) framework^[78]^. A COP is a collaborative group with shared interests that aims to achieve both personal and collective goals, measuring outcomes one to two weeks after completing a 12-week program^[68]^.

Feedback from participants indicated that this combined approach significantly reduced the severity of these disorders among HCPs and increased their overall sense of well-being after a challenging period. The COP framework fosters a supportive network, enhancing treatment flexibility, improving recovery, and decreasing the risk of relapse^[67]^.

Results showed that 91% of patients reported reduced scores and improvement in GAD symptoms, such as constant anxiety, difficulty relaxing, restlessness, irritability, and feelings of impending doom^[68]^. Among depressed patients, 79% experienced lower scores and improvements in appetite, sleep, concentration, negative feelings, and energy^[79]^. By the program’s end, 86% of PTSD screenings were negative, and patients reported overcoming their fears. Additionally, clinical improvement was observed in 92% of patients facing life and work challenges^[68]^.

### Educational programs on ketamine use

Using ketamine at subanesthetic doses may cause pleasurable psychoactive effects such as perceptual disturbances, derealization, depersonalization, altered body perceptions, and impaired proprioception that lead to reactional misuse, especially among males aged 16 to 25 in the USA^[73]^. Some evidence from single-dose studies reported that using 0.5 mg/kg as well as 1.0 mg/kg showed efficacy without superiority of 1.0 mg/kg over 0.5 mg/kg. Treatment-related adverse effects and misuse were found to be affected by higher doses^[46]^. Misuse of ketamine necessitates the cooperation of mental health providers and researchers to find a way to regulate administration so that it is adequate for its desired effect^[80]^.

Recently, there has been a decrease in the number of people who use ketamine as a hallucinogenic drug due to efforts made by governments, training, and courses to prevent drug abuse by creating rehab programs^[81,82]^. A study conducted in 2019 appraised the effect of a brief information, motivation, and behavior skills (IMB) program and an education-as-usual (EAU) program in increasing awareness about ketamine abuse^[83]^. Program evaluation questionnaires classify participants into five groups by asking questions about age, gender, education level, what they know about ketamine, and how to detect the stage of ketamine cessation. Participants were classified as being in the maintenance stage if they had not used ketamine for over six months. Those who used ketamine for more than six months were classified in the action stage. Participants who were considering or planning to stop ketamine use within the next 30 days were in the preparation stage. Those contemplating or planning to stop within the next six months were in the contemplation stage, while those not considering stopping within the next six months were classified as being in the pre-contemplation stage^[83]^.

Additionally, the IBM course is given by a specialized psychiatric team. The IBM program aims to increase the desire for change by speaking about predisposing factors, explaining the most effective refusal skills, showing examples of effective desire control, and right behavior with relapsing sensation. Behavior change occurs by recognizing which behavior needs to be altered primarily with hard efforts and motivation^[83]^. On the other hand, EAU was presented by one lawyer, two MD psychiatrists, one infectious disease MD physician, and one PhD counselor/therapist. EAU aims to warn about the dangerous impact of ketamine on the brain, applicable regulations and laws, and the risks and transmission methods of infectious diseases, such as HIV and hepatitis C^[83]^. According to the study, IBM is more effective at increasing awareness and knowledge about ketamine use and abuse (105).

## Integration of clinical guidelines for standardizing ketamine use

Ketamine’s administration must be performed by certified healthcare professionals, including anesthesiologists, certified nurse anesthetists, pain specialists, emergency physicians, and psychiatrists, to optimize therapeutic outcomes while minimizing adverse events^[84]^. An interprofessional approach is crucial for maximizing the drug’s effectiveness, ensuring that healthcare providers are well-versed in patient eligibility, intravenous (IV) ketamine administration, and potential post-treatment reactions. Structured clinical guidelines provide a framework for standardized ketamine use, particularly for TRD, emphasizing the importance of specific dosing protocols tailored to individual patient needs^[52]^. Initial dosing typically starts at 0.5 mg/kg administered over 40 minutes, with adjustments made based on patient tolerance and weight^[46,85]^.

The recent opioid crisis has sparked renewed interest in ketamine’s analgesic properties, necessitating the development of consistent guidelines to maximize its benefits while mitigating potential psychomimetic and cardiovascular effects^[86,87]^. An SDDK of 0.6 mg/kg has been identified as effective for pain relief without significant psychomimetic side effects. The latest safety protocols stress strict dosing and monitoring practices to reduce the risk of respiratory, cardiovascular, and neuropsychiatric complications, particularly as ketamine is considered a viable alternative to opioids^[53,61]^.

Despite its rapid antidepressant effects, more rigorous empirical research is needed to refine administration protocols and evaluate long-term safety and efficacy across diverse populations. Recent studies, including systematic analyses, indicate that ketamine demonstrates rapid antidepressant effects in both adolescents and older adults, but the quality of evidence remains variable^[88]^. These findings highlight the necessity for well-designed randomized controlled trials to further assess ketamine’s therapeutic potential and optimize treatment outcomes across different demographics.

## Future directions in safety monitoring and management

Advanced safety protocols for ketamine focus on optimizing administration routes (Table 4), such as IN, oral, and IV, while minimizing side effects. Personalized dosing regimens, based on factors like chronic illness and BMI, are crucial for effective treatment^[89]^. Baseline psychiatric status should guide dosing, with lower doses for episodic depression and higher doses for chronic pain. Technological monitoring devices are recommended for early detection of adverse effects, while biomarker profiling offers improved monitoring efficiency. Further research on ketamine’s pharmacodynamics and safer analogs is essential to reduce side effects and misuse risks^[78,90,91]^.

**Table 4:** Different routes of administration of ketamine and associated adverse effects

route	Bioavailability^[94-96]^	Onset of action^[93]^	Duration of action^[93]^	Therapeutic applications^[97-99]^	Safety and adverse effects^[79,100-103]^

	
Intravenous (IV)	100%	Within seconds	30–45 minutes	Induction of anesthesia, sedation, major depressive episodes, chronic pain	increase in blood pressure, perceptual disturbance, drowsiness, dizziness, dissociation and abuse
Intranasal (IN)	45%	5–10 minutes	45–60 minutes	Treatment-related major depressive disorder, chronic pain	Abuse, dissociation feelings, altered state of consciousness, and sensory detachment
					Nasal discomfort, irritation, and rhinorrhea
Rectal	25–30%	10 minutes	30 minutes–2 hours	Pain management, sedation, and in emergency when IV access isn’t possible	Possible irritation or discomfort in the rectal area and abuse
Oral	20%	15–20 minutes	1–2 hours	Sedation, Adjuvant to morphine, and in analgesic	Nausea, vomiting, and dry mouth
					Long-term use has the potential to cause cystitis.
					And abuse
Intramuscular (IM)	93%	1–5 minutes	30 minutes–2 hours	Following acute trauma for analgesia And Sedation	Possible discomfort or pain where the injection is administered
					Strong dissociation, anxiety, and hallucinations
					Increase in heart rate and blood pressure

### Conclusion

Ketamine’s pharmacological benefits span various medical fields, but its safety profile necessitates careful use. Strict adherence to guidelines is essential to mitigate risks like psychological effects, cardiovascular issues, and dependency. With its growing role in psychiatry, further research should focus on dosing optimization, patient monitoring, and long-term use guidelines. Investigating alternative formulations and administration routes may enhance safety. Advancing knowledge of ketamine’s therapeutic window and risks will promote its safe clinical integration.

## Data Availability

**None.**

## Research registration unique identifying number (UIN)

A unique identifying number (UIN) was not required for this review.
